# Maximum parsimony based resolution of inter-species phylogenetic relationships in *Citrus* L. (Rutaceae) using ITS of rDNA

**DOI:** 10.1080/13102818.2014.901665

**Published:** 2014-05-01

**Authors:** Mohamed Hamdy Amar, Ahmed H.M. Hassan, Manosh Kumar Biswas, Ehsan Dulloo, Zong-Zhou Xie, Wen-Wu Guo

**Affiliations:** ^a^ Huazhong Agricultural University, Key Laboratory of Horticultural Plant Biology (MOE), Wuhan, China; ^b^ Desert Research Center, Egyptian Deserts Gene Bank, North Sinai, Egypt; ^c^ Bioversity International, Rome, Italy

**Keywords:** Aurantioideae, *Citrus*, ITS, Rutaceae, ribosomal DNA (rDNA), phylogenetic relationship

## Abstract

The present study aims to analyse phylogenetic relationships, using internal transcribed spacer sequence data of ribosomal DNA (rDNA), across 24 *Citrus* species and close relatives by the evaluation of several parameters such as nucleotide substitution (*r*), nucleotide diversity (π) and the estimated values of transition/transversion bias (*R*). The observed results indicated the presence of a wide divergence pattern of rDNA in subfamily Aurantioideae. Maximum parsimony (MP) analysis inferred divergence pattern in the *Citrus* genus. We observed seven strongly supported clades among the subfamily Aurantioideae. We postulate that the present investigation provides a more robust topology of *Citrus* and its close relatives, which can significantly prove as an additional support to resolve the phylogenetic relationships in *Citrus* genera. Therefore, sequences of noncoding regions should exhibit more phylogenetically informative sites than the coding regions do, which is in accordance with the present study.

## Abbreviations

CAPS*:* cleaved amplified polymorphic sequence
ISSR*:* inter-simple sequence repeat
ITS*:* internal transcribed spacer
K1*:* transition/transversion rate for purine
K2*:* transition/transversion rate for pyrimidine
MCL*:* maximum composite likelihood
MEGA 5*:* Molecular Evolutionary Genetics Analysis version 5
MP*:* maximum parsimony
NCCB*:* National Center of Citrus Breeding
ORF*:* open reading frame
*r:* nucleotide substitution
*R:* estimated values of transition/transversion bias
rDNA*:* ribosomal DNA
SNP*:* single nucleotide polymorphism
π*:* nucleotide diversity
SRAP*:* sequence-related amplified polymorphism
UV*:* ultraviolet

## Introduction

Aurantioideae, a subfamily of family Rutaceae, presents a vast variety of commercially important genera such as *Citrus* and *Fortunella*. Interestingly, the taxonomy of *Citrus* is complex and still the precise number of natural species is unclear, mainly because of the sexually compatible relatives.[1,2] Barrett and Rhodes [3] performed a numerical taxonomy and recommended that there were only three true species within the cultivated *Citrus viz.* Citron (*Citrus medica* L.), Mandarin (*Citrus reticulata* Blanco) and Pummelo (*Citrus grandis* L. Osbeck). The origins of other species are a result of hybridization of these true species. In view of this, taxonomic characterization is critically important for the *Citrus* genus, which has the widest divergence reported among the fruit species and it is imperative to resolve the phylogeny in order to have a better understanding of the complexity of the genus and to develop resources for the proper sustainable development of this genus.

Several earlier attempts have been made to revisit the intra- and inter-species relationships in Aurantioideae,[4–8] which have been previously constrained by restricted taxon representation, using a few inferred sequences such as restriction fragment length polymorphisms (RFLPs), or usage of traditional genetic markers such as isozymes, inter simple sequence repeats (ISSRs) or Randomly Amplified Polymorphic DNA markers (RAPDs) for phylogenetic analysis. Although previous reports exploited the sequence-based approach, these approaches were focused on higher taxonomic levels such as order and family.[9–11] Morpho-taxonomy evaluation, however, has serious limitations in a complex genus like *Citrus*. In *Citrus*, molecular phylogeny at various taxonomic levels has been observed in several earlier studies through application of a wide variety of molecular markers such as SSR,[12] ISSR,[13] SRAP and CAPS-SNP,[14] as well as using chloroplast DNA and rDNA markers.[15,16]

Ribosomal DNA (rDNA) as a source of higher genetic variability has been studied extensively for classification and identification at the generic and infra generic levels in plants.[17,18] Consequently, it has been successfully applied to resolve phylogeny in several models and non-model plant species such as in *Triticum*,[19] *Solanum lycopersicum*,[20] *Oryza sativa*,[21] and closely related species of *Citrus*.[15,17,22] In the present study, we used a comparative as well as a combined approach using several parameters such as nucleotide frequency, nucleotide substitution (*r*), nucleotide diversity (π) and the estimated values of transition/transversion bias (*R*) to provide better and significant understanding of the genetic diversity and phylogenetic relations across 24 studied *Citrus* species and other species related to the genus. The present investigation provides an additional support for resolving the phylogeny of the complex genus *Citrus.*

## Materials and methods

### Plant materials and genomic DNA isolation (gDNA)

Twenty-four genotypes belonging to the genus *Citrus* and species related to it, which includes the following major groups as listed in Table 1, were sampled from the National Center of *Citrus* Breeding (NCCB), Huazhong Agricultural University (HZAU), Wuhan, China. Genomic DNA of *Citrus* cultivars (Table 1) was extracted from fresh leaves following the procedure as previously described elsewhere.[23] The quality and concentration of the DNA samples were checked using a UV-1601 spectrophotometer (Shimadzu, Japan) and a sub-aliquot of the DNA was subsequently diluted to 50 ng/μL for further downstream polymerase chain reaction (PCR) analysis of internal transcribed spacer (ITS) sequences. Both the stock and diluted portions were stored at –20 °C.

**Table 1.** Accession list of *Citrus* and its related species sequenced in this study, BLASTX hits against the GenBank database, similarity score and GenBank accession numbers.

			ITS		
S. No.	Group	Scientific name	Blast	Similarity	GenBank accession no.
1	Sweet orange	*C. sinensis* cv. Valencia	AB456094.1	97%	JN681149
2	Sweet orange	*C. sinensis* cv. Anliu	FJ641933.1	100%	JN681150
3	Sour orange	*C. aurantium* (L.) cv. Daidai	DQ369925.1	99%	JN681151
4	Pummelo hybrid	*C. grandis × C. paradisi* cv. HB pummelo	GQ999538.1	98%	JN681152
5	Grapefruit	*C. paradisi Macf.* cv. Red Marsh grapefruit	FJ641932.1	99%	JN681153
6	Pummelo	*C. grandis (L.) Osbeck* cv. Shatian pummelo	FJ641954.1	99%	JN681154
7	Pummelo	*C. grandis (L.) Osbeck* cv. Guan xi Miyon Pummelo	FJ641953.1	99%	JN681155
8	Pummelo	*C. grandis (L.) Osbeck* cv. Gao Ban Pummelo	FJ980439.1	99%	JN681156
9	Citron	*C. medica var sarcodactylis* cv. Fingered Citron	AB456128.1	100%	JN681157
10	Lemon	*C. limon (L.) Burm. f.* cv. Eureka lemon	DQ369931.1	99%	JN681158
11	Lemon	*C. jambhiri (L.) Burm. f.* cv. Rough lemon	FJ980440.1	99%	JN681159
12	Poncirus	*Poncirus trifoliata* (L) Raf.	DQ369928.1	99%	JN681160
13	Citrange	*C. sinensis × P. trifoliata* cv. Citrange	HM992800.1	98%	JN681161
14	Citrumelo	*C. paradisi × P. trifoliata* cv. Citrumelo	GQ464846.1	100%	JN681162
15	Kumquat	*Fortunella* hindsii Swing. cv. Hongkong Kumquat	FJ641924.1	98%	JN681163
16	Kumquat	*Fortunella crassifolia* Swing. cv. Meiwa Kumquat	AB456108.1	99%	JN681164
17	Navel orange	*C. sinensis* cv. Cara Cara	AB456120.1	99%	JN681165
18	Navel orange	*C. sinensis* cv. Newhall	FJ860066.1	99%	JN681166
19	Mandarin	*Citrus reticulata* Blanco cv. Ponkan	FJ860066.1	99%	JN681167
20	Tangerine	*C. reticulata* Blanco cv. Bendizao	AB456128.1	100%	JN661209
21	Mandarin	*C. unshiu Marc.* cv. Guoqing No.1	AB456058.1	99%	JN661210
22	Mandarin	*C. reticulata × C. paradisi* Macf. cv. Nova (hybrid)	AB456127.1	99%	JN661211
23	Mandarin	*C. reticulata × C. sinensis* (L.) Osbeck cv. Murcott (hybrid)	AB456127.1	99%	JN661212
24	Sweet orange	*C. sinensis* cv. Jincheng	FJ641933.1	93%	JN661213

### PCR amplification and sequencing of the ITS region

In our present research, the entire ITS region (including ITS-1 and ITS-2 of nuclear rDNA and the 5.8S rRNA gene) of rDNA was amplified using the primers ITS1 (5′TCCGTAGGTGAACCTGCGG3′) and (5′TCCTCCGCTTATTGATATGC3′) ITS-4 as previously described.[24] Briefly, each PCR cocktail of 25 μL contained 50 ng of genomic DNA, 0.5 pmol of each primer, 0.2 mmol/L dNTPs, 1 U Taq DNA polymerase (Fermentas, Shenzhen, China), 2.5 μL of 10 times PCR buffer supplied by the manufacturer and about 2.5 mmol/L MgCl_2_. The amplification programme consisted of an initial denaturation step at 94 ºC for 4 min, followed by 35 cycles of 94 ºC for 45 s, 55 ºC for 60 s, 72 ºC for 90 s and a final incubation step of 72 ºC for 7 min. The PCR products obtained were further resolved by electrophoresing 10 μL of the amplified aliquot in a 1.5% agarose gel and were subsequently stained using ethidium bromide and visualized under ultraviolet (UV) light. The PCR fragments were excised and purified from the gel, using an E.Z.N.A® Gel Extraction Kit (Omega Bio-Tek, Inc., Norcross, USA) and were subsequently ligated to a pMD18-T Easy vector as per the manufacturer instructions (TaKaRa, Tokyo, Japan). The ligation product was transformed into *E. coli* DH-5α-competent cells, using ampicillin as a selection marker. Three positive colonies from each clone were selected and sequenced by the Uni-Gene Company (Shanghai, China).

### Sequence editing, alignment and phylogenetic inference

Sequencing chromatograms obtained were analysed and vector sequences were trimmed. In all the sequenced ITS regions, after vector trimming open reading frames (ORFs) were predicted using the National Center for Biotechnology Information (NCBI) Open Reading Frame Finder (http://www.ncbi.nlm.nih.gov/gorf/gorf). The trimmed sequences were further aligned using MUSCLE v3.70+ fix1-2 [25] and the resulting alignments were manually checked. Gaps were retained for further analysis. Nucleotide diversity (π), estimated values of transition/transversion bias (*R*), nucleotide substitutions (*r*) for each nucleotide pair and cluster analysis among the 24 *Citrus* genotypes were estimated using MEGA 5.[26] We further computed the maximum composite likelihood (MCL) estimate of the pattern of nucleotide substitution.[26] For the phylogenetic inference, maximum parsimony (MP) trees were computed using MEGA 5.[26] The bootstrap consensus tree inferred from 500 replicates was taken to represent the evolutionary history of the *Citrus* genus and its related species. In brief, the MP tree was obtained using the Subtree–Pruning–Regrafting (SPR) algorithm with search level 1 in which the initial trees were obtained with the random addition of sequences (10 replicates). The analysis involved 25 nucleotide sequences corresponding to a total of 943 positions in the final dataset. This approach has been previously followed for resolving the phylogeny of Indian *Citrus* cultivars.[17] The phylogenetic tree was re-rooted using the ITS sequence of *Atlantia monophylla* (NCBI accession number, GQ225867) as reported in a previous study.[17]

## Results and discussion

In our study, first after subsequent cleaning of the ITS sequence, we performed homology searches against the NCBI GenBank (https://www.ncbi.nlm.nih.gov/) database, using BLASTn, which revealed 93%–100% similarity with the previously sequenced ITS regions, providing an evidence for the good trustworthiness of ITS regions sequenced in this study. All the ITS sequences of *Citrus* and its relative species have been submitted to GenBank databases (www.ncbi.nlm.nih.gov) and can be accessed under accession numbers as referred in Table 1. In our investigation, the universal primers ITS1 (forward) and ITS4 (reverse) amplified the complete ITS region (ITS1, 5.8S rRNA gene and ITS2) but variation for the ITS regions (650–750 bp) was observed for the individual species; however, the observed length was found similar to the ITS length variation as observed in recently sequenced ITS regions in *Citrus* cultivars [17] and also similar to the large-scale ITS sequences in Brassicacea.[27]

Recently, length variation across ITS regions has also been observed in *Cymbidium* species.[28] The nucleotide composition showed an average of guanine and cytosine (GC) (58.5%) and AT (41.5%) content. The highest number of nucleotides for the ITS sequence was observed in *C. reticulata × C. sinensis* (788 bases), whereas the lowest one was recorded in *C. sinensis* cv. Newhall (609 bases). The maximum GC content (64%) and the lowest AT content (35.8%) were observed in the case of *C. sinensis* cv. Anliu, *C. paradisi* Macf. cv. Red Marsh grapefruit, *Fortunella* hindsii Swing. cv. Hongkong Kumquat, *C. sinensis* cv. Valencia and *C. reticulata × C. sinensis* (L.) Osbeck cv. Murcott (hybrid). Nevertheless, the lowest GC content (46%) and maximum AT content (54%) were recorded in *Poncirus trifoliata* (L) Raf. A similar GC content was observed in the sequenced ITS region of Indian cultivars, which supports the observed pattern of GC variation.[17] In view of the relatively rapid evolution rate, differences in sequence and/or length of ITS rDNA are possible between close species.[22] Sequence length variation in ITS and significant difference in the nucleotide composition were also observed in *Cymbidium* species,[28] which supports our present results.

We further evaluated the nucleotide diversity value (π), using the Tajima Neutrality test.[29] We observed a total of 334 segregating sites (S), 390 maximum number of positions (N) and 24 sites (M) demonstrating a higher nucleotide diversity rate (0.41) among the *Citrus* genus and its closely related species. It is a well-known fact that during DNA sequence evolution, the rate of transitional changes differs quite relatively from the rate of transversional changes, with transitions generally occurring more frequently than transversions. The transition/transversion bias (*R*) across the combined data was evaluated using Kimura two-parameter analysis with four models (K2+G+I, K2+I, K2+G and K2) to describe the best substitution pattern (Table 2). The highest number of substitutions (*r*) for each nucleotide pair was recorded among *r* (CG ±0.189), revealing high levels of substitutions. However, moderate and lower values of substitution were observed for *r* (AG; TC; CT; GA ±0.132) and (AC; TA; TG; CA; GT; GC ±0.059), respectively.[30] The transition/transversion rate ratios observed in our analyses were K1 = 2.136 and K2 = 1.716. However, we observed a higher transition/transversion rate for purine (K1 = 2.136) as compared to recent reports in Indian cultivars (K1 = 1.716) and as compared to the transition/transversion rate for pyrimidine (K2 = 2.796).[17] In our analyses, we observed that the overall transition/transversion bias is *R* = 0.956, which gives a strong support for the dominance of the transitions over transversion in *Citrus* germplasm. The observed higher transition/transversion (*R*) rate is in accordance with the recent reports of the observed higher transition/transversion bias (1.158) in the phylogeny in Indian *Citrus* cultivars recently inferred using ITS.[17] The present rate of transition/transversion bias is also in complete agreement with a recently observed transition bias in *Citrus* germplasm using SSR markers.[1]

**Table 2.** Maximum-likelihood fits, using the Kimura two-parameter model among 24 different nucleotide sequences for combined data of ITS.

Model	Invariant (+*I*)	*R*	Freq A	Freq T	Freq C	Freq G	*r*(AT)	*r*(AC)	*r*(AG)	*r*(TA)	*r*(TC)	*r*(TG)	*r*(CA)	*r*(CT)	*r*(CG)	*r*(GA)	*r*(GT)	*r*(GC)
K2+G+I	0.07	1.14%	0.25	0.25	0.25	0.25	0.058	0.058	0.133	0.058	0.133	0.058	0.058	0.133	0.58	0.133	0.058	0.058
K2+I	0.1	1.11%	0.25	0.25	0.25	0.25	0.059	0.059	0.131	0.059	0.131	0.059	0.059	0.131	0.06	0.131	0.059	0.059
K2+G	0.07	1.15%	0.25	0.25	0.25	0.25	0.058	0.058	0.134	0.058	0.134	0.058	0.058	0.134	0.06	0.134	0.058	0.058
K2	0.1	1.07%	0.25	0.25	0.25	0.25	0.060	0.060	0.129	0.06	0.129	0.06	0.06	0.129	0.06	0.129	0.06	0.06
Average	0.085	1.11%	0.25%	0.25%	0.25%	0.25%	0.059	0.059	0.132	0.059	0.132	0.059	0.059	0.132	0.189	0.132	0.059	0.059

Note: +*I*: evolutionarily invariable; *R*: estimated values of transition/transversion bias; Freq: nucleotide frequencies; and *r*: substitutions for each nucleotide pair.

**Figure 1.**
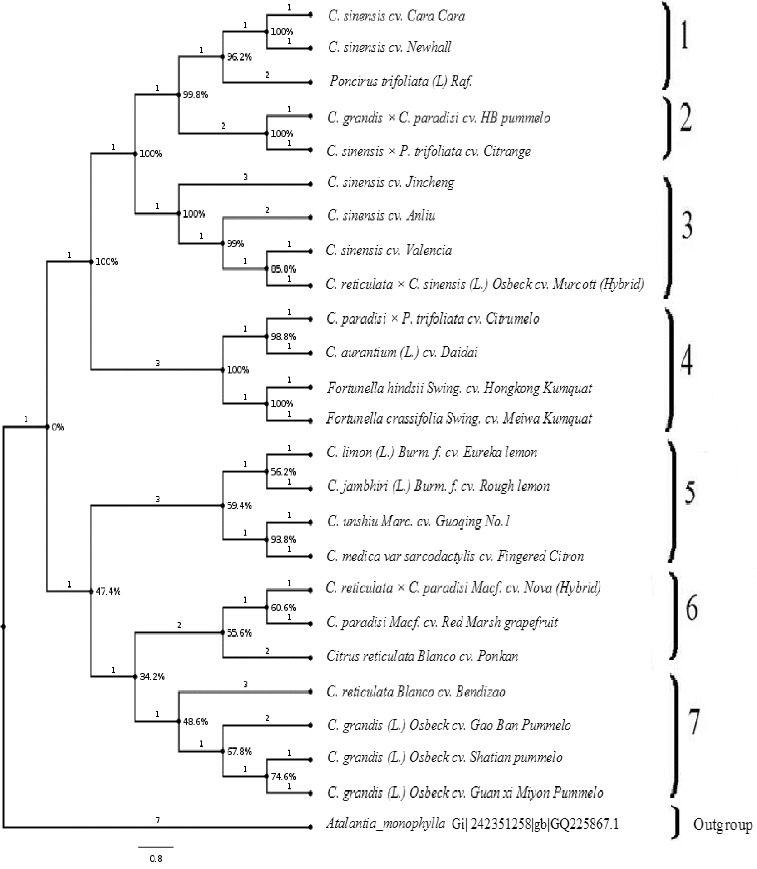
Maximum parsimony analysis of genotypes of the *Citrus* genus and its related species based on ITS rDNA data.

Up to now, there are only a few reports, which sufficiently explain the significance of ITS rDNA as a molecular genotyping tool in *Citrus*.[2,17,22] We analysed the evolutionary history of *Citrus* and its relative species by the MP method, using the SPR algorithm with search level 1 in which the initial trees were obtained by the random addition of sequences (10 replicates) as implemented in MEGA5.[26] The analysis involved 25 nucleotide sequences and there were a total of 943 positions in the final dataset for MP analysis. In the present investigation, using our data, we obtained the most parsimonious tree with a length of 1818 (Figure 1). MP inferred a consistency index (CI – 0.666868), a retention index (RI – 0.792247) and a composite index of 0.551697. The phylogenetic tree was re-rooted using *A. monophylla* (NCBI accession number, GQ225867) as an outgroup species (Figure 1). Recently, *A. monophylla* has been used as an outgroup for inferring the phylogeny in *Citrus* cultivars.[17] In a recent study, using the ITS sequence data and MP analysis, a similar consistency (0.6804) and retention (0.7350) index was observed, which is in line with our observed results and supports the present MP phylogenetic inference.[17]

The phylogenetic analysis revealed several well-supported clades with strong bootstrap values. In total, we observed seven strongly supported clades, which were clearly distinguishable among the subfamily *Aurantioideae*. The first clade was clustered jointly *C. sinensis* cv. Cara Cara, *C. sinensis* cv. Newhall (navel oranges) and *Poncirus trifoliata* with a bootstrap value of 96.2%. In context, the second clade represents the *C. grandis × C. paradisi* cv. HB pummelo and *C. sinensis × P. trifoliata* cv. Citrange. Additionally, *C. sinensis* cv. Jincheng, *C. sinensis* cv. Anliu, *C. sinensis* cv. Valencia and *C. reticulata × C. sinensis* L. *Osbeck* cv. Murcott (hybrid) were placed together in the third clade with bootstrap values of 100%. The phylogenetic analysis, as inferred for the ITS sequence data indicated that sweet oranges (Jincheng, Anliu and Valencia) showed a close relationship with the hybrid Murcott (*C. reticulata × C. sinensis*). The observed clades are in strong support with the previously observed clades.[2,5,15,16,31,32]

The genus *Fortunella* contains the Kumquats. It closely resembles *Citrus* species, although their morphology is very different. Across the fourth clade, *C. paradisi × P. trifoliata* cv. Citrumelo, *C. aurantium* (L.) cv. Daidai, *Fortunella hindsii* Swing. cv. Hongkong Kumquat and *Fortunella crassifolia* Swing. cv. Meiwa Kumquat were grouped in a sister clade. Morphologically, *Fortunella* and *Citrus* are significantly different from each other. However, there are previous evidences of nested clustering of *Fortunella* with *Citrus* species.[8,12] Kyndt et al. [2] demonstrated the inclusion of the *Fortunella* spp. within *Citrus*, close to the *C. reticulata* group, confirming their recent reclassification as *C. japonica*, using ITS sequence polymorphism. Our ITS rDNA data showed a close evolutionary relationship between *Fortunella* (Hongkong and Meiwa Kumquat) and sour orange (*C. aurantium*). Biswas et al. [33] reported that *Fortunella* might be less divergent than *Citrus* at the molecular level than observed in morphology. Moreover, *C. medica* var *sarcodactylis* cv. Fingered Citron, *C. unshiu* Marc. cv. Guoqing No.1, *C. jambhiri* (L.) Burm. f. cv. Rough lemon and *C. limon* (L.) Burm. f. cv. Eureka lemon occupies the fifth clade. Several earlier experts hypothesized *C. limon* to have a complex hybrid origin of Citron and Lime [34–36] or Citron and Sour Orange [7,37] or Sour Orange and Lime.[38,39] In our study, *C. limon* was grouped with *C. jambhiri* and *C. medica* var *sarcodactylis* in the MP tree, which supports the close relationship among these species. In a previous study by Jena et al.,[15] they proposed a close relationship between *C. jambhiri* and *C. reticulata* and supported the role of *C. reticulata* as a maternal parent in the hybrid origin of *C. jambhiri*, which perfectly fits with the phylogenetically observed clade in our analyses.

The citron mitotype contained only *C. medica*. This species did not transmit its cytoplasm to other species but played an important role as a male parent.[32] Indeed, our results confirmed that citron was grouped with *C. jambhiri* (L.) Burm. f. cv. Rough lemon and *C. limon* (L.) Burm. f. cv. Eureka Lemon. Furthermore, *Citrus reticulata Blanco* cv. Ponkan, *C. paradisi Macf.* cv. Red Marsh grapefruit and *C. reticulata × C. paradisi Macf.* cv. Nova (hybrid) were grouped jointly as a sixth clade. As it is well known, the origin of grapefruit has been well documented and is considered to have originated most probably from a hybrid between pummelo and sweet orange, perhaps through back introgression to pummelo.[5,40] In our study, the ITS rDNA data indicated that *C. paradisi Macf.* cv. Red Marsh grapefruit and the *C. reticulata × C. paradisi Macf.* cv. Nova (hybrid) were grouped together with pummelo, supporting the viewpoint of a backcross with pummelo.[8] For *C. grandis*, *C. grandis* L. *Osbeck* cv. Guan xi Miyon Pummelo, *C. grandis* L. *Osbeck* cv. Gao Ban pummelo and *C. grandis* L. *Osbeck* cv. Shatian pummelo were grouped with *C. reticulata Blanco* cv. Bendizao in the last clade. In addition, Shatian pummelo, Gao Ban pummelo and Guan xi Miyon Pummelo (C*. grandis*) were clustered with *C*. *reticulata* (Ponkan mandarin). In contrast, HB pummelo (*C. grandis* × *C. paradisi*) is closer to Citrange (*C. sinensis × P. trifoliata*). It is generally accepted that citrons, mandarin and pummelo are three true species in the genus *Citrus*.[33,40] Our data inferred a close genetic relationship between mandarin and pummelo, concordant with the previous results of Xu et al.,[22] which supports this theory, as mandarin and pummelo each had a near uniform ITS rDNA sequence. In this context, Froelicher et al. [32] strongly proposed that mandarin played an important role in the evolution of cultivated *Citrus*; in addition to this, the authors supported that pummelo mitotype was found to be present as the most important cultivated *Citrus* species. Furthermore, Barkley et al. [12] reported that it was a mixture between the citron, mandarin and pummelo groups with the majority of its alleles coming from the citron and mandarin groups.

## Conclusions

To conclude, the present study presents an effective utilization of rDNA sequence divergence to maximize the possible knowledge of the genetic diversity within the *Citrus* genus and its relatives. The phylogenetic tree of the rDNA supported seven strong clades which were clearly shown among the genus *Citrus*. Consequently, this study not only corroborated the previous molecular reconstruction of subfamily *Aurantioideae*, but also strengthened previous claims concerning the evolutionary biology of the genus *Citrus*.
